# Diguanidinium bis­(μ-2-hydroxy­propane-1,2,3-tricarboxyl­ato)bis­[diaqua­zincate(II)] dihydrate

**DOI:** 10.1107/S1600536809054439

**Published:** 2010-01-09

**Authors:** Mohammad T. M. Al-Dajani, Hassan H. Abdallah, Nornisah Mohamed, Chin Sing Yeap, Hoong-Kun Fun

**Affiliations:** a School of Pharmaceutical Sciences, Universiti Sains Malaysia, 11800 USM, Penang, Malaysia; b School of Chemical Sciences, Universiti Sains Malaysia, 11800 USM, Penang, Malaysia; c X-ray Crystallography Unit, School of Physics, Universiti Sains Malaysia, 11800 USM, Penang, Malaysia

## Abstract

The asymmetric unit of the title compound, (CH_6_N_3_)_2_[Zn_2_(C_6_H_5_O_7_)_2_(H_2_O)_2_]·2H_2_O, contains one-half of a centrosymmetric dizinc(II) complex anion, one guanidinium cation and one water mol­ecule. Each Zn^II^ ion is hexa­coordinated by two citrate anions, one in a bidentate fashion and the second monodentate, and two water mol­ecules in a distorted octa­hedral geometry. Intra­molecular O—H⋯O hydrogen bonds add further stability to the mol­ecular structure. In the crystal structure, mol­ecules are linked into a three-dimensional framework by inter­molecular N—H⋯O, O—H⋯O and C—H⋯O hydrogen bonds.

## Related literature

For general background to guanidine and citric acid, see: Raczyńska *et al.* (2003 ▶); Yamada *et al.* (2009 ▶); Sigman *et al.* (1993 ▶); Schuck (1934 ▶); Sherman *et al.* (1936 ▶). For applications of citric acid in industry and materials science, see: Blair *et al.* (1991 ▶); Jiang *et al.* (2007 ▶). For related guanidinium structures, see: Al-Dajani *et al.* (2009**a* ▶,b* ▶). For hydrogen-bond motifs, see: Bernstein *et al.* (1995 ▶).
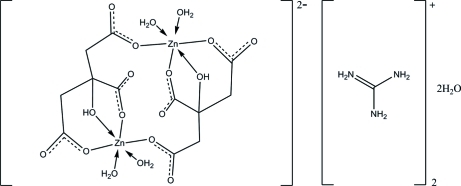


## Experimental

### 

#### Crystal data

(CH_6_N_3_)_2_[Zn_2_(C_6_H_5_O_7_)_2_(H_2_O)_2_]·2H_2_O
*M* *_r_* = 737.21
Monoclinic, 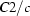

*a* = 28.9405 (4) Å
*b* = 8.5708 (1) Å
*c* = 11.3395 (2) Å
β = 95.249 (1)°
*V* = 2800.89 (7) Å^3^
*Z* = 4
Mo *K*α radiation
μ = 1.81 mm^−1^
*T* = 296 K
0.32 × 0.30 × 0.18 mm

#### Data collection

Bruker SMART APEXII CCD area-detector diffractometer
Absorption correction: multi-scan (*SADABS*; Bruker, 2005 ▶) *T*_min_ = 0.593, *T*_max_ = 0.734
32332 measured reflections
7693 independent reflections
5495 reflections with *I* > 2σ(*I*)
*R*_int_ = 0.026

#### Refinement

*R*[*F* ^2^ > 2σ(*F* ^2^)] = 0.031
*wR*(*F* ^2^) = 0.084
*S* = 1.05
7693 reflections
190 parameters
H-atom parameters constrained
Δρ_max_ = 0.45 e Å^−3^
Δρ_min_ = −0.29 e Å^−3^

### 

Data collection: *APEX2* (Bruker, 2005 ▶); cell refinement: *SAINT* (Bruker, 2005 ▶); data reduction: *SAINT*; program(s) used to solve structure: *SHELXTL* (Sheldrick, 2008 ▶); program(s) used to refine structure: *SHELXTL*; molecular graphics: *SHELXTL*; software used to prepare material for publication: *SHELXTL* and *PLATON* (Spek, 2009 ▶).

## Supplementary Material

Crystal structure: contains datablocks global, I. DOI: 10.1107/S1600536809054439/sj2710sup1.cif

Structure factors: contains datablocks I. DOI: 10.1107/S1600536809054439/sj2710Isup2.hkl

Additional supplementary materials:  crystallographic information; 3D view; checkCIF report

## Figures and Tables

**Table 1:** Hydrogen-bond geometry (Å, °)

*D*—H⋯*A*	*D*—H	H⋯*A*	*D*⋯*A*	*D*—H⋯*A*
O3—H1*O*3⋯O6	0.91	1.87	2.6445 (13)	142
N1—H1*N*1⋯O1*W*	0.86	2.38	3.1607 (19)	150
N1—H2*N*1⋯O4	0.92	2.10	2.9855 (17)	161
N2—H1*N*2⋯O5^i^	0.88	2.12	2.9351 (18)	154
N2—H2*N*2⋯O1*W*^ii^	0.88	2.05	2.9077 (19)	166
N3—H1*N*3⋯O4^i^	0.86	2.29	3.1011 (18)	156
N3—H1*N*3⋯O5^i^	0.86	2.55	3.3221 (18)	150
N3—H2*N*3⋯O1	0.85	2.36	3.1788 (18)	162
O1*W*—H1*W*1⋯O2^iii^	0.80	1.97	2.7640 (15)	177
O1*W*—H2*W*1⋯O5^iv^	0.82	2.05	2.8568 (17)	170
O2*W*—H1*W*2⋯O1^iii^	0.84	1.88	2.7172 (12)	171
O2*W*—H2*W*2⋯O7^v^	0.76	1.86	2.6116 (13)	167
O3*W*—H1*W*3⋯O2^iii^	0.85	1.90	2.7215 (12)	163
O3*W*—H2*W*3⋯O7^vi^	0.74	1.92	2.6424 (12)	166
C5—H5*B*⋯O3*W*^vii^	0.97	2.53	3.4943 (13)	172

Symmetry codes: (i) 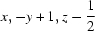; (ii) 
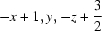
; (iii) 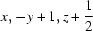; (iv) 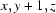; (v) 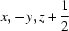; (vi) 
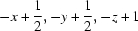
; (vii) 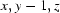.

**Fig. 1.**
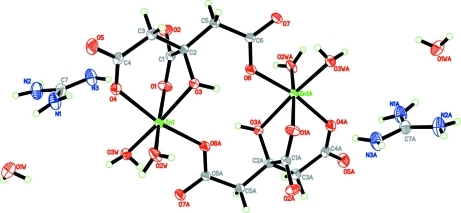
The molecular structure of the title compound with atom labels and 30% probability ellipsoids for non-H atoms. Atoms with suffix A are generated by the symmetry operation (1/2 - x, 1/2 - y, 1 - z).

**Fig. 2.**
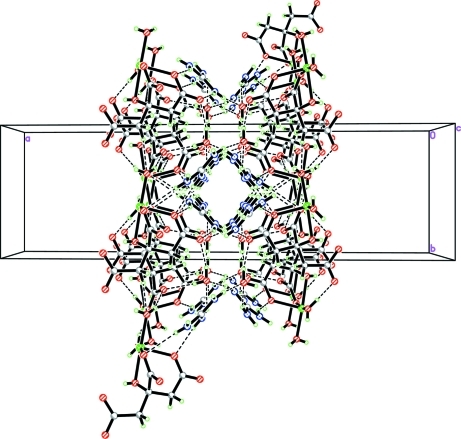
The crystal packing of title compound, viewed down the c axis, showing the hydrogen-bonded (dashed lines) three-dimensional framework. Hydrogen atoms not involved in the hydrogen-bonding have been omitted for clarity.

## supplementary crystallographic information

### Comment

Citric acid or 2-hydroxy-1,2,3-propanetricarboxylic acid contains three carboxyl groups.  It is found in the literature that an organism has the ability to synthesize citric acid. (Schuck, 1934; Sherman *et al.*, 1936). Citric acid has many applications including use in the manufacture of detergents, shampoos, cosmetics and in chemical cleaning (Blair *et al.*, 1991). It can also be used for the preparation of the catalyst LaNiO_3_ for the preparation of carbon nanotubes (Jiang *et al.*, 2007).

Guanidine can be formed by the oxidation of guanine as a final product of the protein metabolism (Raczyńska *et al.*, 2003; Yamada *et al.*, 2009; Sigman *et al.*, 1993).

The asymmetric unit of title compound contains one half of a dizinc(II) complex anion, one guanidinium cation and one water solvent molecule (Fig. 1). The anion lies across a crystallographic inversion center, the other half is symmetry generated [symmetry code: 1/2 - *x*, 1/2 - *y*, 1 - *z*]. The Zn1 and Zn2 ions are coordinated to four O atoms from two citrate anions and two water molecules to form a distorted octahedral geometry. Two citric acid molecules are deprotonated and two guanidine molecules protonated to yield the cation and anion. The geometrical parameters of the guanidinium cations agree with those previously reported (Al-Dajani *et al.*, 2009*a,b*). An intramolecular O3—H1O3···O6 hydrogen bond generates an *S*(6) ring motif (Bernstein *et al.*, 1995).

In crystal structure (Fig. 2), the Zn^II^ complex anion and water molecules are linked into sheets parallel to the *bc* plane *via* intermoleculer O1W—H1W1···O2, O1W—H2W1···O5, O2W—H1W2···O1, O2W—H2W2···O7, O3W—H1W3···O2, O3W—H2W3···O7 and C5—H5B···O3W hydrogen bonds. The guanidinium cations are linked these sheets generating a three-dimensional framework through N—H···O hydrogen bonds (Table 1).

### Experimental

Citric acid (anhydrous) (0.02 mol, 3.85 g) was dissolved in THF in a flat bottom flask with magnetic stirrer. In a separating funnel, guanidine carbonate (0.005 mol, 0.9 g), 99% [H_2_NC(NH)NH_2_].2H_2_CO_3_ was dissolved in THF. The guanidine solution was added in small portions to the citric acid with stirring. At room temperature, zinc chloride (ZnCl_2_) (0.02 mol, 2.75 g) was added also with stirring. The reaction mixture was refluxed for 30 min. After cooling the mixture to room temperature, it was left stirring overnight. The resulting colourless crystals were filtered, washed with methanol and dried at 353 K.

### Refinement

N-bound and O-bound H atoms were located in a difference Fourier map and refined as riding on their parent atom, with *U*_iso_(H) = 1.2, 1.5*U*_eq_(N, O). The remaining H atoms were positioned geometrically [C–H = 0.97 Å and refined using a riding model, with *U*_iso_(H) = 1.2*U*_eq_(C)].

### Crystal data

**Table:**

(CH_6_N_3_)_2_[Zn_2_(C_6_H_5_O_7_)_2_(H_2_O)_2_]·2H_2_O	*F*(000) = 1520
*M**_r_* = 737.21	*D*_x_ = 1.748 Mg m^−^^3^
Monoclinic, *C*2/*c*	Mo *K*α radiation, λ = 0.71073 Å
Hall symbol: -C 2yc	Cell parameters from 9983 reflections
*a* = 28.9405 (4) Å	θ = 2.5–34.8°
*b* = 8.5708 (1) Å	µ = 1.81 mm^−^^1^
*c* = 11.3395 (2) Å	*T* = 296 K
β = 95.249 (1)°	Block, colourless
*V* = 2800.89 (7) Å^3^	0.32 × 0.30 × 0.18 mm
*Z* = 4	

### Data collection

**Table:**

Bruker SMART APEXII CCD area-detector diffractometer	7693 independent reflections
Radiation source: fine-focus sealed tube	5495 reflections with *I* > 2σ(*I*)
graphite	*R*_int_ = 0.026
φ and ω scans	θ_max_ = 38.3°, θ_min_ = 2.5°
Absorption correction: multi-scan (*SADABS*; Bruker, 2005)	*h* = −48→50
*T*_min_ = 0.593, *T*_max_ = 0.734	*k* = −14→12
32332 measured reflections	*l* = −19→16

### Refinement

**Table:**

Refinement on *F*^2^	Primary atom site location: structure-invariant direct methods
Least-squares matrix: full	Secondary atom site location: difference Fourier map
*R*[*F*^2^ > 2σ(*F*^2^)] = 0.031	Hydrogen site location: inferred from neighbouring sites
*wR*(*F*^2^) = 0.084	H-atom parameters constrained
*S* = 1.05	*w* = 1/[σ^2^(*F*_o_^2^) + (0.0404*P*)^2^ + 0.5068*P*] where *P* = (*F*_o_^2^ + 2*F*_c_^2^)/3
7693 reflections	(Δ/σ)_max_ = 0.002
190 parameters	Δρ_max_ = 0.45 e Å^−^^3^
0 restraints	Δρ_min_ = −0.29 e Å^−^^3^

### Special details

**Table:**

Geometry. All e.s.d.'s (except the e.s.d. in the dihedral angle between two l.s. planes) are estimated using the full covariance matrix. The cell e.s.d.'s are taken into account individually in the estimation of e.s.d.'s in distances, angles and torsion angles; correlations between e.s.d.'s in cell parameters are only used when they are defined by crystal symmetry. An approximate (isotropic) treatment of cell e.s.d.'s is used for estimating e.s.d.'s involving l.s. planes.
Refinement. Refinement of *F*^2^ against ALL reflections. The weighted *R*-factor *wR* and goodness of fit *S* are based on *F*^2^, conventional *R*-factors *R* are based on *F*, with *F* set to zero for negative *F*^2^. The threshold expression of *F*^2^ > σ(*F*^2^) is used only for calculating *R*-factors(gt) *etc*. and is not relevant to the choice of reflections for refinement. *R*-factors based on *F*^2^ are statistically about twice as large as those based on *F*, and *R*- factors based on ALL data will be even larger.

### Fractional atomic coordinates and isotropic or equivalent isotropic displacement parameters (Å^2^)

**Table:**

	*x*	*y*	*z*	*U*_iso_*/*U*_eq_	
Zn1	0.322092 (4)	0.394890 (14)	0.631912 (11)	0.02481 (4)	
O1	0.33231 (4)	0.37163 (9)	0.45220 (8)	0.03439 (19)	
O2	0.36716 (3)	0.20991 (10)	0.33603 (8)	0.03573 (18)	
O3	0.31089 (3)	0.15045 (9)	0.60117 (7)	0.02494 (14)	
H1O3	0.2815	0.1428	0.5651	0.037*	
O4	0.39328 (3)	0.33281 (11)	0.66307 (9)	0.03675 (19)	
O5	0.44700 (3)	0.16069 (13)	0.72462 (10)	0.0474 (2)	
O6	0.24882 (3)	0.07623 (10)	0.42434 (9)	0.0365 (2)	
O7	0.25557 (3)	−0.16794 (10)	0.36365 (10)	0.0427 (2)	
C1	0.34724 (4)	0.23880 (12)	0.42612 (9)	0.02427 (18)	
C2	0.34175 (3)	0.10406 (10)	0.51507 (9)	0.02214 (16)	
C3	0.38848 (4)	0.06848 (13)	0.58412 (10)	0.0288 (2)	
H3A	0.3844	−0.0221	0.6334	0.035*	
H3B	0.4101	0.0393	0.5275	0.035*	
C4	0.41101 (4)	0.19654 (14)	0.66293 (10)	0.0295 (2)	
C5	0.32367 (4)	−0.04392 (12)	0.44989 (10)	0.0273 (2)	
H5A	0.3410	−0.0586	0.3814	0.033*	
H5B	0.3302	−0.1325	0.5020	0.033*	
C6	0.27248 (4)	−0.04513 (12)	0.40863 (10)	0.02705 (19)	
C7	0.45073 (5)	0.64963 (16)	0.50364 (12)	0.0382 (3)	
N1	0.45132 (5)	0.61025 (16)	0.61676 (12)	0.0511 (3)	
H1N1	0.4644	0.6641	0.6751	0.061*	
H2N1	0.4393	0.5187	0.6424	0.061*	
N2	0.47821 (5)	0.76043 (17)	0.47097 (13)	0.0552 (3)	
H1N2	0.4783	0.7888	0.3966	0.066*	
H2N2	0.5000	0.7945	0.5235	0.066*	
N3	0.42259 (5)	0.57429 (17)	0.42351 (13)	0.0547 (3)	
H1N3	0.4208	0.6209	0.3559	0.066*	
H2N3	0.4020	0.5135	0.4454	0.066*	
O1W	0.46013 (4)	0.85648 (15)	0.82510 (11)	0.0525 (3)	
H1W1	0.4335	0.8373	0.8306	0.079*	
H2W1	0.4593	0.9470	0.8020	0.079*	
O2W	0.31354 (4)	0.38177 (10)	0.80498 (8)	0.0388 (2)	
H1W2	0.3163	0.4610	0.8492	0.058*	
H2W2	0.2975	0.3236	0.8321	0.058*	
O3W	0.33513 (3)	0.63182 (9)	0.63707 (8)	0.03042 (16)	
H1W3	0.3494	0.6661	0.7004	0.046*	
H2W3	0.3106	0.6561	0.6328	0.046*	

### Atomic displacement parameters (Å^2^)

**Table:**

	*U*^11^	*U*^22^	*U*^33^	*U*^12^	*U*^13^	*U*^23^
Zn1	0.02665 (6)	0.02218 (6)	0.02509 (7)	0.00090 (4)	−0.00047 (4)	−0.00195 (4)
O1	0.0534 (5)	0.0230 (3)	0.0269 (4)	0.0080 (3)	0.0047 (4)	0.0038 (3)
O2	0.0384 (4)	0.0412 (4)	0.0285 (4)	0.0094 (4)	0.0079 (3)	0.0043 (3)
O3	0.0251 (3)	0.0245 (3)	0.0252 (3)	−0.0002 (3)	0.0025 (3)	−0.0024 (3)
O4	0.0276 (4)	0.0324 (4)	0.0490 (5)	−0.0006 (3)	−0.0035 (3)	−0.0064 (4)
O5	0.0344 (5)	0.0509 (6)	0.0527 (6)	0.0026 (4)	−0.0192 (4)	−0.0009 (5)
O6	0.0275 (4)	0.0277 (4)	0.0521 (5)	0.0073 (3)	−0.0089 (4)	−0.0145 (4)
O7	0.0306 (4)	0.0291 (4)	0.0666 (7)	0.0027 (3)	−0.0057 (4)	−0.0205 (4)
C1	0.0243 (4)	0.0252 (4)	0.0226 (4)	0.0018 (3)	−0.0016 (3)	0.0015 (3)
C2	0.0221 (4)	0.0207 (4)	0.0231 (4)	0.0036 (3)	−0.0010 (3)	−0.0007 (3)
C3	0.0259 (5)	0.0282 (4)	0.0307 (5)	0.0052 (4)	−0.0055 (4)	0.0016 (4)
C4	0.0226 (4)	0.0355 (5)	0.0298 (5)	−0.0006 (4)	−0.0011 (4)	0.0022 (4)
C5	0.0246 (4)	0.0220 (4)	0.0345 (5)	0.0042 (3)	−0.0010 (4)	−0.0040 (4)
C6	0.0262 (4)	0.0242 (4)	0.0302 (5)	0.0032 (4)	−0.0005 (4)	−0.0051 (4)
C7	0.0341 (6)	0.0395 (6)	0.0399 (7)	−0.0022 (5)	−0.0028 (5)	0.0025 (5)
N1	0.0547 (8)	0.0579 (8)	0.0388 (7)	−0.0176 (6)	−0.0058 (6)	0.0040 (5)
N2	0.0531 (7)	0.0576 (8)	0.0534 (8)	−0.0190 (6)	−0.0043 (6)	0.0139 (6)
N3	0.0586 (8)	0.0594 (8)	0.0436 (7)	−0.0202 (7)	−0.0091 (6)	0.0033 (6)
O1W	0.0347 (5)	0.0645 (7)	0.0585 (7)	0.0026 (5)	0.0055 (5)	0.0105 (6)
O2W	0.0571 (6)	0.0320 (4)	0.0284 (4)	−0.0179 (4)	0.0093 (4)	−0.0048 (3)
O3W	0.0321 (4)	0.0257 (3)	0.0332 (4)	−0.0030 (3)	0.0009 (3)	−0.0003 (3)

### Geometric parameters (Å, °)

**Table:**

Zn1—O2W	2.0036 (9)	C3—H3B	0.9700
Zn1—O3W	2.0653 (8)	C5—C6	1.5123 (15)
Zn1—O1	2.0953 (9)	C5—H5A	0.9700
Zn1—O6^i^	2.1071 (9)	C5—H5B	0.9700
Zn1—O4	2.1259 (9)	C7—N2	1.3135 (18)
Zn1—O3	2.1436 (8)	C7—N1	1.3250 (19)
O1—C1	1.2624 (12)	C7—N3	1.3305 (18)
O2—C1	1.2430 (13)	N1—H1N1	0.8653
O3—C2	1.4382 (12)	N1—H2N1	0.9161
O3—H1O3	0.9125	N2—H1N2	0.8782
O4—C4	1.2757 (15)	N2—H2N2	0.8767
O5—C4	1.2389 (14)	N3—H1N3	0.8616
O6—C6	1.2669 (12)	N3—H2N3	0.8455
O6—Zn1^i^	2.1071 (9)	O1W—H1W1	0.7969
O7—C6	1.2498 (13)	O1W—H2W1	0.8189
C1—C2	1.5510 (14)	O2W—H1W2	0.8432
C2—C3	1.5302 (14)	O2W—H2W2	0.7641
C2—C5	1.5355 (14)	O3W—H1W3	0.8481
C3—C4	1.5237 (16)	O3W—H2W3	0.7377
C3—H3A	0.9700		
			
O2W—Zn1—O3W	93.79 (3)	C2—C3—H3B	107.8
O2W—Zn1—O1	171.26 (3)	H3A—C3—H3B	107.2
O3W—Zn1—O1	94.55 (3)	O5—C4—O4	122.94 (11)
O2W—Zn1—O6^i^	95.68 (4)	O5—C4—C3	116.42 (11)
O3W—Zn1—O6^i^	93.63 (3)	O4—C4—C3	120.64 (9)
O1—Zn1—O6^i^	86.38 (4)	C6—C5—C2	115.82 (8)
O2W—Zn1—O4	91.59 (4)	C6—C5—H5A	108.3
O3W—Zn1—O4	94.00 (4)	C2—C5—H5A	108.3
O1—Zn1—O4	85.24 (4)	C6—C5—H5B	108.3
O6^i^—Zn1—O4	169.08 (3)	C2—C5—H5B	108.3
O2W—Zn1—O3	94.25 (3)	H5A—C5—H5B	107.4
O3W—Zn1—O3	171.92 (3)	O7—C6—O6	123.51 (10)
O1—Zn1—O3	77.38 (3)	O7—C6—C5	117.91 (9)
O6^i^—Zn1—O3	86.39 (3)	O6—C6—C5	118.54 (9)
O4—Zn1—O3	84.96 (3)	N2—C7—N1	120.18 (13)
C1—O1—Zn1	113.29 (7)	N2—C7—N3	120.45 (14)
C2—O3—Zn1	106.65 (6)	N1—C7—N3	119.36 (13)
C2—O3—H1O3	106.7	C7—N1—H1N1	124.8
Zn1—O3—H1O3	105.3	C7—N1—H2N1	123.6
C4—O4—Zn1	127.79 (7)	H1N1—N1—H2N1	111.5
C6—O6—Zn1^i^	125.31 (7)	C7—N2—H1N2	121.7
O2—C1—O1	124.51 (10)	C7—N2—H2N2	117.8
O2—C1—C2	117.97 (9)	H1N2—N2—H2N2	119.7
O1—C1—C2	117.48 (9)	C7—N3—H1N3	111.4
O3—C2—C3	106.41 (8)	C7—N3—H2N3	120.1
O3—C2—C5	110.47 (8)	H1N3—N3—H2N3	124.1
C3—C2—C5	109.15 (8)	H1W1—O1W—H2W1	102.7
O3—C2—C1	110.03 (7)	Zn1—O2W—H1W2	121.5
C3—C2—C1	110.05 (9)	Zn1—O2W—H2W2	124.3
C5—C2—C1	110.64 (9)	H1W2—O2W—H2W2	108.4
C4—C3—C2	117.86 (9)	Zn1—O3W—H1W3	116.0
C4—C3—H3A	107.8	Zn1—O3W—H2W3	95.9
C2—C3—H3A	107.8	H1W3—O3W—H2W3	110.5
C4—C3—H3B	107.8		
			
O3W—Zn1—O1—C1	150.43 (8)	O1—C1—C2—O3	12.55 (13)
O6^i^—Zn1—O1—C1	−116.20 (9)	O2—C1—C2—C3	73.57 (12)
O4—Zn1—O1—C1	56.79 (8)	O1—C1—C2—C3	−104.39 (11)
O3—Zn1—O1—C1	−29.10 (8)	O2—C1—C2—C5	−47.13 (12)
O2W—Zn1—O3—C2	−143.52 (7)	O1—C1—C2—C5	134.91 (10)
O1—Zn1—O3—C2	33.95 (6)	O3—C2—C3—C4	−56.32 (12)
O6^i^—Zn1—O3—C2	121.05 (6)	C5—C2—C3—C4	−175.54 (10)
O4—Zn1—O3—C2	−52.29 (6)	C1—C2—C3—C4	62.86 (12)
O2W—Zn1—O4—C4	91.34 (10)	Zn1—O4—C4—O5	−150.02 (10)
O3W—Zn1—O4—C4	−174.75 (10)	Zn1—O4—C4—C3	30.55 (16)
O1—Zn1—O4—C4	−80.50 (10)	C2—C3—C4—O5	174.68 (11)
O6^i^—Zn1—O4—C4	−40.5 (3)	C2—C3—C4—O4	−5.86 (17)
O3—Zn1—O4—C4	−2.78 (10)	O3—C2—C5—C6	45.40 (12)
Zn1—O1—C1—O2	−160.08 (9)	C3—C2—C5—C6	162.07 (10)
Zn1—O1—C1—C2	17.74 (12)	C1—C2—C5—C6	−76.69 (12)
Zn1—O3—C2—C3	84.83 (8)	Zn1^i^—O6—C6—O7	−4.75 (18)
Zn1—O3—C2—C5	−156.82 (6)	Zn1^i^—O6—C6—C5	177.29 (8)
Zn1—O3—C2—C1	−34.36 (9)	C2—C5—C6—O7	−174.40 (11)
O2—C1—C2—O3	−169.49 (9)	C2—C5—C6—O6	3.68 (16)

Symmetry codes: (i) −*x*+1/2, −*y*+1/2, −*z*+1.

### Hydrogen-bond geometry (Å, °)

**Table:**

*D*—H···*A*	*D*—H	H···*A*	*D*···*A*	*D*—H···*A*
O3—H1O3···O6	0.91	1.87	2.6445 (13)	142
N1—H1N1···O1W	0.86	2.38	3.1607 (19)	150
N1—H2N1···O4	0.92	2.10	2.9855 (17)	161
N2—H1N2···O5^ii^	0.88	2.12	2.9351 (18)	154
N2—H2N2···O1W^iii^	0.88	2.05	2.9077 (19)	166
N3—H1N3···O4^ii^	0.86	2.29	3.1011 (18)	156
N3—H1N3···O5^ii^	0.86	2.55	3.3221 (18)	150
N3—H2N3···O1	0.85	2.36	3.1788 (18)	162
O1W—H1W1···O2^iv^	0.80	1.97	2.7640 (15)	177
O1W—H2W1···O5^v^	0.82	2.05	2.8568 (17)	170
O2W—H1W2···O1^iv^	0.84	1.88	2.7172 (12)	171
O2W—H2W2···O7^vi^	0.76	1.86	2.6116 (13)	167
O3W—H1W3···O2^iv^	0.85	1.90	2.7215 (12)	163
O3W—H2W3···O7^i^	0.74	1.92	2.6424 (12)	166
C5—H5B···O3W^vii^	0.97	2.53	3.4943 (13)	172

Symmetry codes: (ii) *x*, −*y*+1, *z*−1/2; (iii) −*x*+1, *y*, −*z*+3/2; (iv) *x*, −*y*+1, *z*+1/2; (v) *x*, *y*+1, *z*; (vi) *x*, −*y*, *z*+1/2; (i) −*x*+1/2, −*y*+1/2, −*z*+1; (vii) *x*, *y*−1, *z*.
